# Hybrid Integrated Wearable Patch for Brain EEG-fNIRS Monitoring

**DOI:** 10.3390/s24154847

**Published:** 2024-07-25

**Authors:** Boyu Li, Mingjie Li, Jie Xia, Hao Jin, Shurong Dong, Jikui Luo

**Affiliations:** Key Laboratory of Advanced Micro/Nano Electronic Devices & Smart Systems of Zhejiang, College of Information Science and Electronic Engineering, Zhejiang University, Hangzhou 310027, China; 12331032@zju.edu.cn (B.L.);

**Keywords:** co-located, EEG-fNIRS, noise suppression, crosstalk suppression, acquisition module design, acquisition module mechanical design

## Abstract

Synchronous monitoring electroencephalogram (EEG) and functional near-infrared spectroscopy (fNIRS) have received significant attention in brain science research for their provision of more information on neuro-loop interactions. There is a need for an integrated hybrid EEG-fNIRS patch to synchronously monitor surface EEG and deep brain fNIRS signals. Here, we developed a hybrid EEG-fNIRS patch capable of acquiring high-quality, co-located EEG and fNIRS signals. This patch is wearable and provides easy cognition and emotion detection, while reducing the spatial interference and signal crosstalk by integration, which leads to high spatial–temporal correspondence and signal quality. The modular design of the EEG-fNIRS acquisition unit and optimized mechanical design enables the patch to obtain EEG and fNIRS signals at the same location and eliminates spatial interference. The EEG pre-amplifier on the electrode side effectively improves the acquisition of weak EEG signals and significantly reduces input noise to 0.9 μV_rms_, amplitude distortion to less than 2%, and frequency distortion to less than 1%. Detrending, motion correction algorithms, and band-pass filtering were used to remove physiological noise, baseline drift, and motion artifacts from the fNIRS signal. A high fNIRS source switching frequency configuration above 100 Hz improves crosstalk suppression between fNIRS and EEG signals. The Stroop task was carried out to verify its performance; the patch can acquire event-related potentials and hemodynamic information associated with cognition in the prefrontal area.

## 1. Introduction

Electroencephalogram (EEG) and functional near-infrared spectroscopy (fNIRS) dual-modal synchronous brain signal monitoring systems can accurately and continuously measure the neuronal electrical signal of the surface area and hemodynamic activity of the brain deep area. It combines the advantages of the high spatial resolution of fNIRS and high temporal resolution of EEG to provide a comprehensive picture of brain function [1]. EEG-fNIRS systems have been applied across various fields of brain science. Clinically, EEG-fNIRS systems have been proven to provide important diagnostic information for the evaluation or treatment of stroke [2], seizure [3], and Alzheimer’s disease [4], among other diseases [5,6]. In the field of brain–computer interfaces (BCIs) [7,8], the EEG-fNIRS system has been utilized to fabricate a hybrid BCI (hBCI) to improve classification accuracy [9,10]. To better study the spatiotemporal associations between the hemodynamic–electrical patterns of brain functions and further improve the classification and decoding accuracy of BCIs, co-located EEG-fNIRS signals attract attention because of their high spatial and temporal coupling and adaptation to tight time synchronization requirements [11].

**Figure 1:**
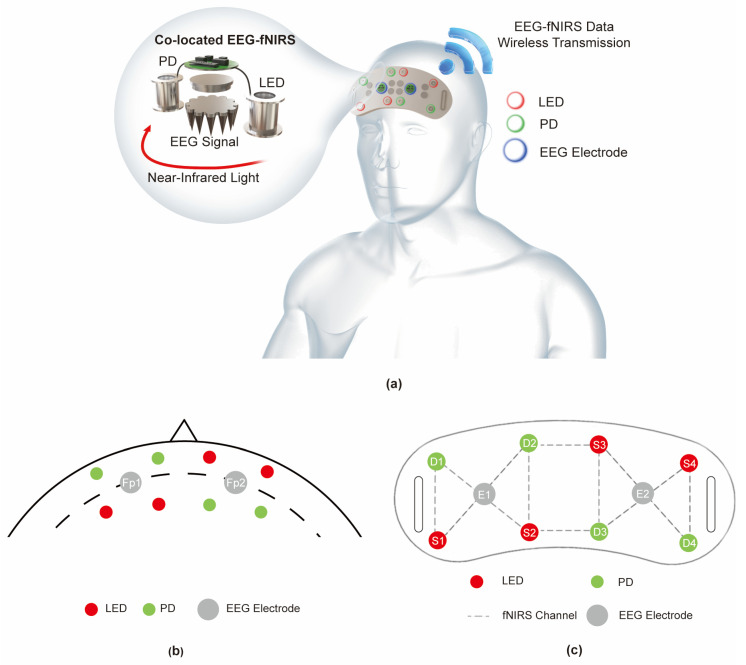
(**a**) Overall system architecture. (**b**) Layout of EEG and fNIRS sensors. (**c**) Positioning structure of EEG electrodes, LEDs, and PDs.

In order to obtain functional imaging of EEG and fNIRS simultaneously, many discrete or integrated EEG-fNIRS systems or ICs have been developed, such as discrete commercial EEG systems and fNIRS systems and combined EEG-fNIRS system [12], NIRS/EEG monitoring of ASIC [13], and modular hybrid systems [14]. However, acquiring co-located EEG-fNIRS signals still remains a challenge due to the spatial interference between the EEG and fNIRS acquisition modules, signal crosstalk between EEG-fNIRS signals, and signal synchronization problems. Especially, as shown Figure 1, the prefrontal cortex region, which is related to cognition and emotions, needs to be monitored via simultaneous EEG and fNIRS signals in a limited area.

In this article, we report an integrated EEG-fNIRS patch with a novel circuit architecture and optimized acquisition module design, which can achieve two-channel EEG and ten-channel fNIRS measurements simultaneously. The patch achieves synchronized, low-noise, and low-crosstalk EEG-fNIRS acquisition by integrating the following features and structures.

- EEG-fNIRS acquisition module design and optimized mechanical design enables the acquisition module to obtain EEG and fNIRS signals at the same location and eliminates spatial interference, while increasing the scalability of the patch.
- EEG pre-amplifier design is utilized on the electrode side for EEG preprocessing, which can effectively improve weak EEG signal acquisition and noise suppression.
- ADS1299- and AFE4404-based analog front-end (AFE) architecture is designed, which achieves synchronous, high-resolution EEG and fNIRS signal measurements.
- Crosstalk between fNIRS signals and EEG signals is minimized through above 100 Hz high LED switching frequency configuration.

Several evaluation tests were performed to verify the co-located EEG-fNIRS hybrid data acquisition performance. We demonstrate that the patch performs with low input noise (0.9 V_rms_), low frequency distortion (<1%), and low amplitude distortion (<2%). Based on these ideal properties, we show that the developed patch can acquire event-related potentials and hemodynamic information at prefrontal areas in the event-related Stroop task. Our approach provides a step towards highly coupled spatial and temporal EEG-fNIRS signal acquisition, laying the foundation for the comprehensive exploration of brain functional activity.

## 2. Materials and Methods

### 2.1. Overall System Architecture

The overall system architecture is shown in Figure 1a. This patch was used to support the co-located EEG-fNIRS signal acquisition in the forehead, and provides synchronous, low-noise, and low-crosstalk dual-mode signal acquisition while realizing integration and wireless data transmission. As shown in Figure 1b, according to the international 10–20 system, two EEG electrodes were placed at Fp1 and Fp2. Four optical sources and four optical detectors were located over the prefrontal area around Fp1, Fpz, and Fp2.

As shown in Figure 1c, in order to acquire neuronal activity from the same location, the EEG electrode was placed in the middle between the source (LED) and the detector (PD), so as to achieve the same channel configuration [15]. An LED was used as the light source because it can be directly attached to the scalp without fiber cables, which greatly increases the flexibility of the acquisition module layout. Each LED can provide 1 fNIRS channel, which has the same acquisition location as the EEG channel. And, 4 fNIRS channels were placed at the same acquisition location as the EEG channel. This patch can provide a total of 10 fNIRS channels and 2 EEG channels, in which 4 fNIRS channels are at the same acquisition location as the EEG channel. The patch can measure the EEG and fNIRS signals at Fp1 and Fp2 simultaneously while covering the active frontal brain regions as much as possible [16], which can support the monitoring needs of cerebral hemodynamic response and EEG response in depressive disorder, cognitive event classification, and other cognitive or emotional tasks [17,18].

### 2.2. System Design

**Figure 2:**
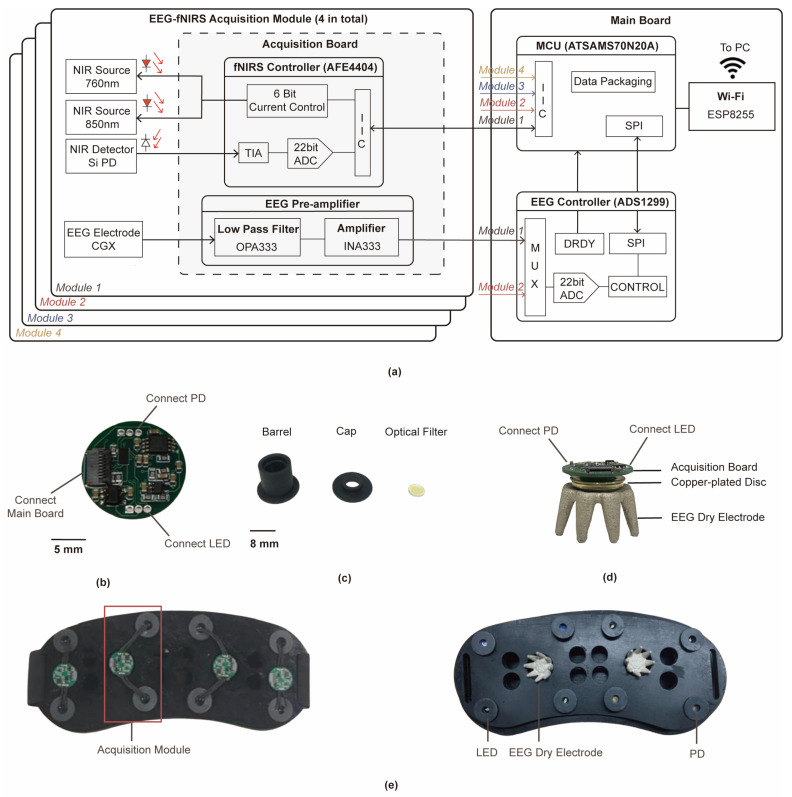
(**a**) The system circuit block diagram. (**b**) The proposed acquisition board. (**c**) The barrel, cap, and optical filter to fix the LED and PD. (**d**) The EEG-fNIRS acquisition module of the proposed patch. (**e**) Front and back view of the entire patch.

EEG and fNIRS signals are highly sensitive to noise and prone to crosstalk. Therefore, the hardware architecture illustrated in Figure 2a has been designed to improve small-amplitude EEG signals acquisition, noise, and crosstalk suppression, which is in concordance with the system concept of “co-located EEG and fNIRS acquisition”. As is shown in Figure 2a, EEG electrodes, LEDs, and detectors were integrated into separate EEG-fNIRS acquisition modules. This allows the monitoring range to be extended to the whole brain by simply adding EEG-fNIRS acquisition modules. The patch implements in this paper contains 4 EEG-fNIRS acquisition modules and 1 main board.

The acquired EEG signal was firstly processed by the EEG pre-circuit on the EEG-fNIRS acquisition module illustrated in Figure 2a. The EEG pre-circuit included a two-stage filter and amplifier circuit. High-frequency noise was filtered out using an OPA333-based active low-pass filter with a cutoff frequency of 50 Hz. 

An INA333-based signal amplifier was employed to provide a voltage gain of 1000 *v*/*v* (60 dB), which was capable of improving the acquisition performance of small amplitude EEG signals and providing high input impedance (100 GΩ). EEG signals from multiple acquisition modules were fed into ADS1299 in parallel, and the multiplexer in ADS1299 allows low-crosstalk, multi-channel synchronous input without sampling and holding circuits, which improved the integration of the patch. Digitized by a 24-bit resolution ADC, the EEG signals were transmitted to MCU via an SPI bus. The module can acquire EEG signals at a sampling rate up to 16 kSPS.

The acquired bio-optical signal was input into AFE4404 on the acquisition module, converted into a voltage signal by an integrated transimpedance amplifier (TIA), and then digitized by an integrated 24-bit analog-to-digital converter (ADC). The high dynamic range (100 dB) enables an excellent signal to noise ratio (SNR), even for small amplitude bio-optical signals in the presence of large signal artifacts. The ADC data were subsequently transmitted to the micro controller unit (MCU) via an IIC bus. The module can acquire fNIRS signals at a sampling rate up to 100 Hz. The switching time between the two measured wavelengths was controlled by the “Data Ready” pin (DRDY) of ADS1299 to ensure that synchronization between EEG and bio-optical signals can be obtained even if there are errors in the reference clocks of the two AFEs.

As shown in Figure 2d, the LEDs and PDs were are connected to the acquisition board by wire, and a copper-plated disk was used to connect the EEG electrodes and the acquisition board to form an EEG-fNIRS acquisition module.

The whole system was embedded in a framework made up of an ATSAMS70N20A (Microchip) MCU on the main board. A detailed diagram of the signal processing workflow can be found in Figures S1 and S2. The MCU will send the packetized EEG and fNIRS data to the PC via the external ESP8285 module for further processing. Please see Note S1 for details of the data processing flow in the PC. 

LEDs of 760 nm and 850 nm dual-wavelengths (Ushio epitex L760_850−04A) were used for fNIRS light sources. Each LED adopted wavelength time division multiplexing. Silicon photodiodes (Hamamatsu S5972) were used for fNIRS detectors. The PD exhibited high photoelectric sensitivity (>0.5 A/W) at both 760 nm and 850 nm while having the features of small size, low power dissipation, and a high level of noise suppression. As shown in Figure 2c, the fNIRS light sources and detectors were fixed using a probe. Each probe is consisted of a circular filter (LP900), a 3D-printed cap, and a 3D-printed barrel. The circular filter uses long-wave pass filter, which meets the high transmittance of emitted light at 760 nm and 850 nm while filtering out ambient light interference signals.

A claw-shaped dry electrode (CGX) was used for EEG acquisition [19]. The electrode was small in size, easy to install, and the surface was plated with a Ag/AgCl layer, which helped to realize miniaturization and high integration, overcoming the problem of signal quality degradation and the discomfort of the participants in continuous EEG acquisition based on traditional wet electrodes. The dry electrode can support continuous high-quality acquisition for a long time (>30 min) and provides high user comfort and reusability.

Considering the wearing comfortability and convenience of the participant, 3D printing was used to make the fixing belt shown in Figure 2e. The fixing belt was made of thermoplastic polyurethane (TPU), which has good flexibility and flexibility, and ensured that the EEG dry electrodes, LEDs, and PDs closely fit the skin on the forehead.

### 2.3. System Crosstalk Analysis and Suppression

The co-located dual-modal signal acquisition patch will introduce crosstalk between the dual-modal signals. In fact, for example, the instantaneously high current in fNIRS light source driving circuit can easily distort small-amplitude EEG and bio-optical signals [20]. A previous study also showed that switching of NIRS channels may cause high-amplitude noise in the same frequency of EEG, which would cause misjudgment of real neural activity [21]. Therefore, when designing an integrated EEG-fNIRS system, crosstalk between EEG signals and fNIRS signals must be taken into account. In our proposed patch, hardware architecture and software configuration were carefully designed to minimize crosstalk between the dual-modal signals.

To minimize crosstalk between fNIRS signals and EEG signals, first, the LED current switching frequency of the dual-wavelength LED current was configured to be >100 Hz, which far exceeds the EEG frequency band of interest (0–50 Hz), so the crosstalk related to the EEG signal could be clearly separated using a low-pass filter with a cutoff frequency of 50 Hz. Second, integrated EEG AFE circuits on the main board also provided higher crosstalk suppression performance for EEG signals by current path optimization and shielding optimization.

The crosstalk between the EEG signal and fNIRS signal was also minimized by a separate ground design on the acquisition board, ensuring electrical isolation of the EEG and fNIRS signals.

## 3. Evaluation and Experimental Procedure

### 3.1. Evaluation of EEG Acquisition Performance

**Figure 3:**
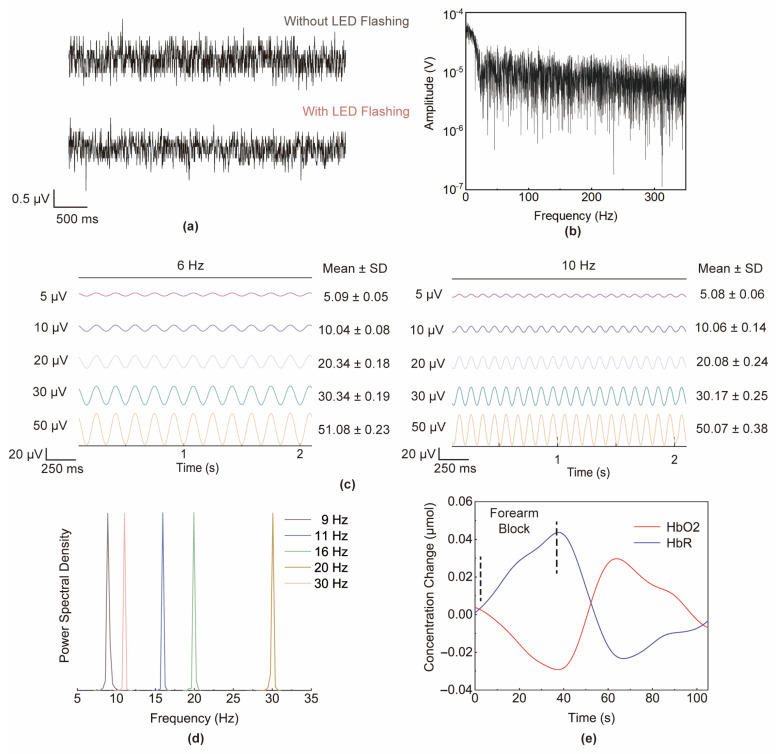
(**a**) EEG input-referred noise in no-LED flashing condition and LED flashing condition; (**b**) EEG input-referred noise spectrum in LED flashing condition; (**c**) EEG amplitude distortion measurement; (**d**) EEG frequency distortion measurement; and (**e**) ΔHbO_2_, ΔHbR trend in forearm block experiment.

We first evaluated the input-referred noise of the EEG acquisition circuit in the LED flashing condition and in the no-LED flashing condition. As shown in Figure 3a, it can be found that in the absence of LED flashing, the input-referred noise was 0.81 μV_rms_. Even with the LED flashing condition, an input-referred noise of 0.89 μV_rms_ was measured and no fNIRS crosstalk component was observed in the spectrum in Figure 3b. These results show that the proposed patch has an excellent noise suppression performance of less than 0.9 μV_rms_. In addition, we evaluated the amplitude distortion and frequency distortion of the acquired EEG signals. As shown in Figure 3c,d, the amplitude distortion and the frequency distortion were less than 2% and less than 1%, respectively. The results verify that the measured EEG signals have low frequency distortion and amplitude distortion. The EEG acquisition module is capable of obtaining high-quality EEG signals. More details about the evaluation experiment can be found in Note S2.

### 3.2. Evaluation of fNIRS Acquisition Performance

Referring to the experiment in [20,22], a forearm block experiment was performed to verify the performance of fNIRS acquisition. The experiment was carried out in a quiet laboratory with no strong light interference. The participants put their arm flat on the table with palm facing up and the wristband was tied to the participant’s forearm. fNIRS light sources and detectors were attached to the participant’s forearm.

We obtained the ΔHbO_2_ and ΔHbR values by analyzing the fNIRS data and the results are plotted in Figure 3d. When the wristband sphygmomanometer was inflated, ΔHbO_2_ dropped slowly and ΔHbR rose slowly due to blood blockage in the forearm. When the wristband sphygmomanometer was deflated and the forearm blood flow was released again, ΔHbO_2_ and ΔHbR dramatically changed toward the baseline, overshooting occurred, and then they gradually converged to the baseline. The experimental results can be mutually verified with the results of the previous forearm blocking experiment [20], indicating that the patch can effectively collect changes in human hemodynamics.

### 3.3. Event-Related Stroop Task

To further validate the ability to acquire the co-located EEG-fNIRS signals, referring to the experiment in [22], an event-related Chinese character Stroop task was designed. The experimental paradigm was used to induce conflicts in cognitive psychology and the activation in participant’s prefrontal cortex can be assessed by EEG and fNIRS signals.

**Figure 4:**
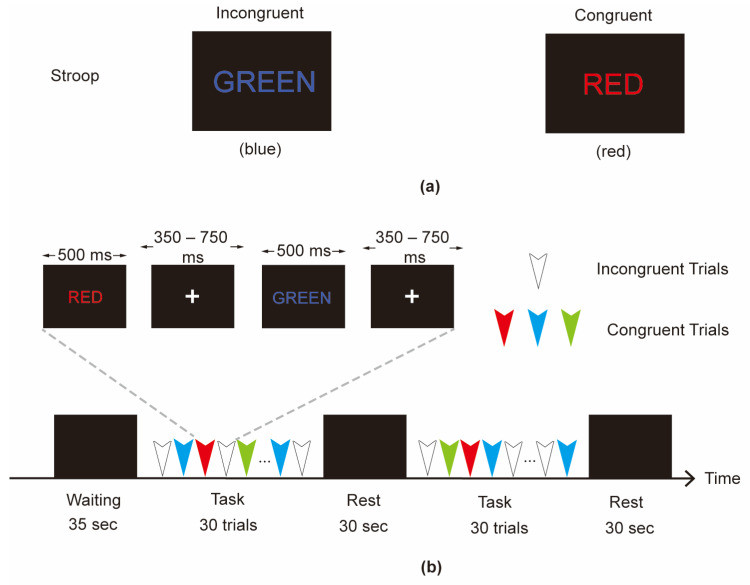
(**a**) Schematic diagram of incongruent and congruent trial. (**b**) Experimental paradigm for Stroop task.

As shown in Figure 4a, the stimuli consisted of a Chinese character with the same or different color and meaning. Under the interference of the meaning of the character, the participants were instructed to judge the color of the Chinese character and press the corresponding key on the keyboard with the right index finger within the time limit. Each task comprised 30 trials, with on-third of trials being congruent (the color and meaning coincided, e.g., the character means “Red” printed in the color red) and two-thirds of trials being incongruent (the word and color did not coincide, e.g., the character means “Red” printed in the color green). The congruent trial and incongruent trial were administered randomly. Each trial was displayed for 500 ms, with a randomly selected interval of 350–750 ms between trials. A detailed experimental design for the event-related Stroop task can be found in Note S3.

The experimental paradigm flow used in this study is shown in Figure 4b. The experimental paradigm was divided into a waiting period, task period, and rest period. During the task period, participants were asked to perform the Stroop task. During the waiting period and rest period, the participants were asked to remain in a relaxed state.

Thirteen healthy volunteers (right-handed, native Chinese speakers, aged 20–29 years; four women and nine men) participated in this experiment. All participants had normal or corrected-to-normal vision, normal color vision, and normal cognitive function. Each participant was seated on an adjustable chair in a sound- and light-attenuated room. The PC monitor was placed 65 cm in front of the participant’s eyes. As shown in Figure 1a, the acquisition module of the EEG-fNIRS patch was worn on the forehead of the participant to acquire EEG signals and fNIRS signals at Fp1 and Fp2. Prior to the formal experiment, participants were asked to run eight trials to make sure they were familiar with the experimental process and could respond correctly. During the experiment, the patch collected EEG signals and fNIRS signals at a sampling rate of 1 kHz and 100 Hz, respectively.

The original EEG signal was analyzed using MATLAB 2023a. Epochs were extracted ranging from −250 ms before to 750 ms after stimulus onset, and baseline signal from −250 ms to 0 ms were corrected. After that, the averaged event-related potentials (ERPs) were band-pass filtered with a cut-off frequency of 0.8 Hz to 17 Hz. On the basis of ERP data, three feature-based components, P450 (positive component from 400 to 450 ms), N500 (negative component from 450 to 550 ms), and P600 (positive component from 600 to 700 ms), were measured at Fp1 and Fp2.

**Figure 5:**
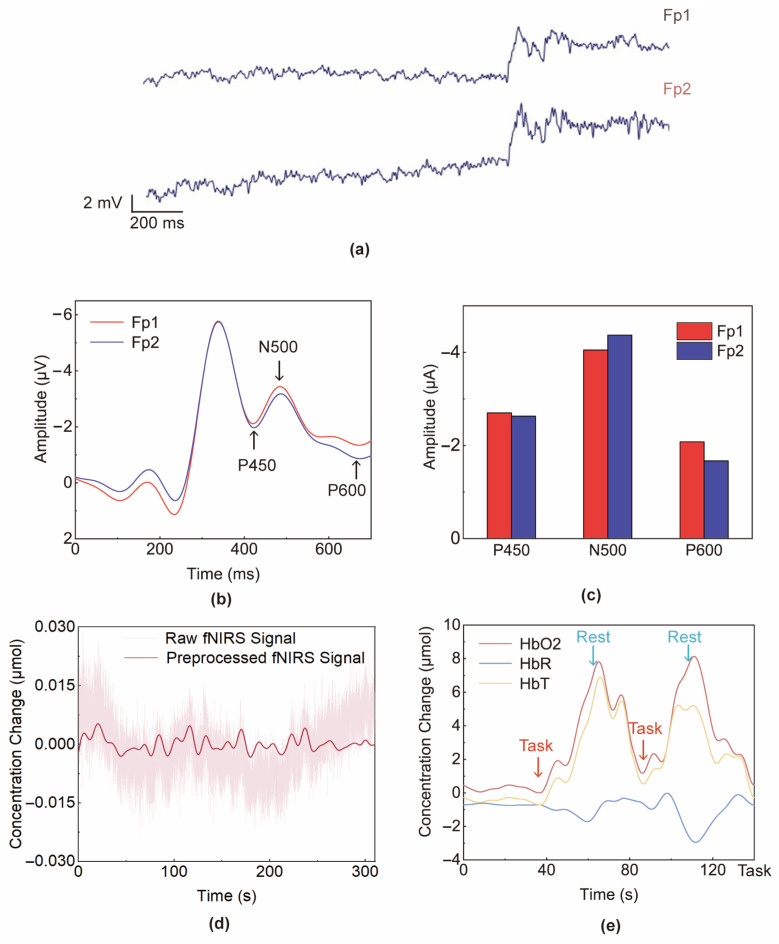
(**a**) Raw EEG data of Fp1 and Fp2; (**b**) ERP results in a trial; (**c**) average amplitude of three ERP components in Fp1 and Fp2; (**d**) comparison of original fNIRS signal and preprocessed fNIRS signal; and (**e**) the ΔHbO2, ΔHbR, and ΔHbT values of the brain Fp1 point in the Stroop task.

Figure 5a shows the raw EEG data of Fp1 and Fp2. And, the ERP results from a trial are shown in Figure 5b. Figure 5c shows the average amplitude of three ERP components in Fp1 and Fp2 across all trials. The amplitude of P450 was −2.70 ± 0.14 and −2.63 ± 0.16 μV (Mean ± SD) in Fp1 and Fp2, respectively, while the amplitude of N500 component was −4.05 ± 0.20 and −4.37 ± 0.25 μV (Mean ± SD) in Fp1 and Fp2, respectively. And, the amplitude of P600 was −2.06 ± 0.27 and −1.67 ± 0.19 μV (Mean ± SD) in Fp1 and Fp2, respectively. Repeated measure analysis of variance (ANOVA) indicated statistically significant differences between the N500 component at the right prefrontal cortex and left prefrontal cortex (*p* = 0.03 < 0.05) and the P600 component at the right prefrontal cortex and left prefrontal cortex (*p* = 0.042 < 0.05). N500 had a stronger response at Fp2, and compared to right prefrontal cortex, P600 had a stronger response at Fp1. The P450 component at the right prefrontal cortex and left prefrontal cortex were not significantly different from each other (*p* = 0.31 > 0.05). The results shows that three ERP components activated in the forehead, which is consistent with the experimental phenomena in the previous literature obtained by the proposed patch [23].

Event-related fNIRS signals are highly susceptible to interference from physiological noise (e.g., 0.2–0.3 Hz respiration component, ~1 Hz heartbeat component, and ~0.1 Hz Mayer waves component) and motion artifacts [24]. Therefore, detrending, motion correction and band-pass filtering are used to remove physiological noise, baseline drift, and motion artifacts from the original fNIRS signal. As shown in Figure 5d, firstly, the modified Beer–Lambert law was utilized to calculate the concentration changes of oxygenated hemoglobin (HbO_2_), deoxygenated hemoglobin (HbR), and total hemoglobin (HbT) according to the calculation method in previous studies [25]. Then, a first-order polynomial regression model was used to remove linear detrends and a temporal derivative distribution repair (TDDR)-based motion correction function was used to remove both spike artifacts and baseline shifts. Considering the hemodynamic response after neural activation embedded in 0.03–0.1 Hz [26], a third-order band-pass IIR filter with a cut-off frequency of 0.01 Hz to 0.08 Hz was applied to remove physiological noise components. 

The ΔHbO_2_, ΔHbR, and ΔHbT values of the brain Fp1 point and Fp2 point collected in fNIRS channels D1-S2 and D4-S3 (shown in Figure 1c), respectively, are shown in Figure 5e. During the waiting period, the concentrations of both HbO_2_ and HbR remained stable. When the first Stoop task began, the HbO_2_ concentration increased rapidly. At the cessation of the task and entry into the rest period, the HbO_2_ concentration gradually returned to the baseline level. During this process, the concentration of HbR showed a roughly opposite trend to the change in the concentration of HbO_2_, which is consistent with the analysis of the mechanism of neural–vascular coupling. It can also be found that the change in the concentration of HbR is smaller than the change in the concentration of HbO_2_. From the perspective of brain activity, when the Stroop task starts, there is an increase in the oxygen demand of the prefrontal cortex involved in cognitive activity, which primarily leads to an increase in cerebral arterial blood flow and dominates the changes in local blood oxygen concentration, and this leads to an increase in HbT. In arterial blood, the proportion of HbO_2_ is higher, which leads to a higher increase in the concentration of HbO_2_ than in the proportion of HbR. Additionally, Pearson correlation analysis showed that the peak amplitude of P600 at Fp1 had a strong correlation with the second peak value of ΔHbR (r = 0.752, *p* = 0.009 < 0.01). The peak amplitude of N500 at Fp1 also had a significant correlation with the first peak value of ΔHbR (r = 0.724, *p* = 0.012). However, peak amplitudes of P450 were not found to correlate with any peak of ΔHbR or ΔHbO_2_. Therefore, a linear regression model can be established using the peak amplitude of P600 and N500 and peak values of ΔHbR to represent the hemodynamic–electrical patterns of brain functions. These conclusions are in good agreement with the findings in [23].

The co-located EEG and fNIRS signals in the Stroop task were effectively detected by our proposed patch, providing brain activation information such as the ERP response and trend in the ΔHbO_2_, ΔHbR response. A conclusion can be drawn that the proposed EEG-fNIRS patch was capable of acquiring neuroelectric and hemodynamic responses at the same location.

## 4. Conclusions

In this study, a two-channel EEG and ten-channel fNIRS hybrid EEG-fNIRS brain monitoring patch has been proposed that can measure EEG and brain cerebral hemodynamic information at the same location. As shown in Table 1, compared with previous research, the proposed EEG-fNIRS acquisition module design and optimized acquisition module layout can acquire co-located EEG-fNIRS signals while eliminating spatial location interference, which can also easily extend the acquisition range to the whole brain. The EEG pre-amplifier on the electrode side effectively provided a high EEG signal noise suppression capability of less than 0.9 μV_rms_, low-amplitude distortion to less than 2%, and low-frequency distortion to less than 1%. Moreover, high LED switching frequency configuration greatly reduces the high crosstalk between bio-optical signals and EEG signals. In addition, detrending, motion correction, and band-pass filter design effectively removed physiological noise, baseline drift, and motion artifacts, effectively improving the SNR. The forearm block experiment and Stroop task showed that the system is sufficiently capable for acquiring neuronal electrical signal and hemodynamic activity at the same location. The small size (about 78.54 mm^2^) and lightweight (about 21.8 g) EEG-fNIRS acquisition module, EEG dry electrodes, and TPU flexible fixing belt can ensure long-term monitoring and wearing comfort to meet the co-located EEG-fNIRS acquisition needs of emotional or cognitive tasks or patients with mild cognitive impairment and major depressive disorder in the home or clinic. It is expected to provide new information and phenomena that cannot be detected when EEG and fNIRS are measured at separate locations, offering richer data for the comprehensive exploration of brain functional activities and introducing new signal acquisition methods for EEG-fNIRS research.

A limitation of our proposed system is that our EEG and fNIRS channels were limited and only covered the forehead, compared to discrete commercial EEG systems, fNIRS systems, and combined EEG-fNIRS system. Although our highly scalable acquisition module design can quickly extend the acquisition range to the whole brain, the low SNR caused by hair absorption and occlusion still limits its application in motor imagery, visual stimulation, and other clinical applications where hemodynamic measurements are required in parietal, occipital, or temporal lobe regions. In addition, a newly designed fixing belt is also needed to ensure that EEG measurements conform to the international 10–20 system. However, the current system has met our design goal of using a wearable, portable patch that allows high-quality acquisition of co-located EEG-fNIRS signals to support cognitive and emotional measurements at the prefrontal lobe. Therefore, our next steps should focus on how to reconstruct fNIRS signals impaired by extra-cranial confounds using both algorithms and hardware approaches to improve the usability of the system for brain–computer interfaces and brain research. 

## Figures and Tables

**Table 1:** Comparison of features between the proposed and previous EEG-fNIRS systems.

System	[13]	[14]	[20]	[27]	[28]	Our Work
EEG input-referred noise	1.21 μV_rms_	1.39 μV_pp_	0.14 μV_rms_	29.9 μV_rms_	0.44 μV_rms_	0.89 μV_rms_
EEG sampling rate	-	16 kSPS	250 Hz	250 SPS	2 kSPS	16 kSPS
fNIRS sampling rate	512 SPS	500 SPS	5 Hz	8 SPS	10 Hz	100 Hz
EEG resolution	15	24	24	24	12	24
fNIRS resolution	-	24	16	24	12	24
Dry EEG electrode	No	No	Yes	No	Yes	Yes
Co-located EEG/fNIRS acquisition	Yes	No	No	No	No	Yes
Crosstalk suppression	No	Yes	Yes	Yes	No	Yes
fNIRS physiological noise removal	16 Hz Low-pass filter	-	RC Low-pass filter	Low-pass filter	-	0.01–0.08 Hz Band-pass filter
fNIRS detrending	-	-	Baseline correction	-	-	First-order polynomial regression
fNIRS motion artifacts removal	-	-	-	-	-	TDDR

- This parameter is not provided in the reference.

## Data Availability

The source data and source code for this article are available on GitHub. Please visit: https://github.com/Shirakami114514/Hybrid-integrated-wearable-patch-for-brain-EEG-fNIRS-monitoring (accessed on 22 July 2024).

## Supplementary Materials

The following supporting information can be downloaded at: https://www.mdpi.com/article/10.3390/s24154847/s1, Figure S1: EEG signal processing workflow. Figure S2: fNIRS signal processing workflow. Note S1: EEG and fNIRS data processing process in PC. Note S2: EEG acquisition performance evaluation method. Note S3: Experimental design for the event-related Stroop task.
