# Supplementary material for: Hybrid Integrated Wearable Patch for Brain EEG-fNIRS Monitoring
Source: Sensors (Basel). 2024 Jul 25;24(15):4847. doi: 10.3390/s24154847 (PMC11314658; doi:10.3390/s24154847)
Supplement: Supplementary file 1 [file sensors-24-04847-s001.zip › sensors-3024661-supplementary.pdf]

Figure S1. EEG signal processing workflow

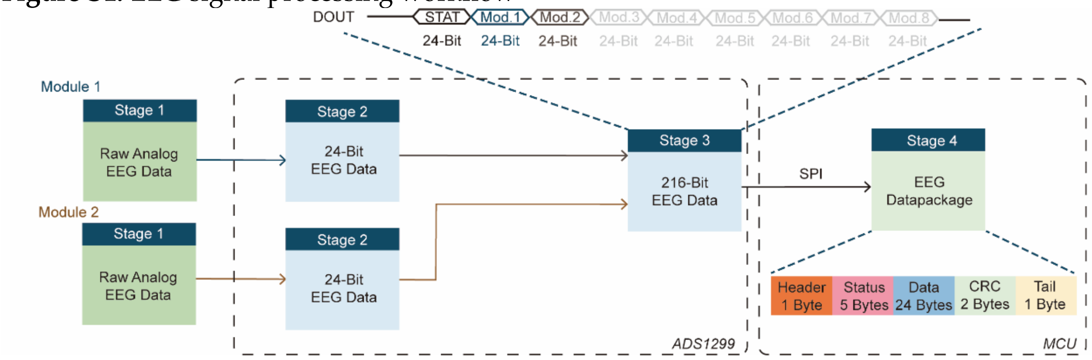

The raw EEG signal obtained through the EEG preamplifier circuit is first digitized into a 24 Bit EEG signal by the 24 Bit ADC integrated in the ADS1299. According to the SPI protocol of ADS1299, the data from the SPI bus will be transferred to the MCU in the format of 'status data + 8-channel EEG data' with a total of 216 Bit (the current proposed system uses the data of the first 2 channels). The MCU will packetize the EEG data, where the 1 Byte 'Header' and the 1 Byte 'Tail' are used to delimit the packets, the 5 Bytes 'Status' are used to indicate the packet serial number, packet length and data type, in addition to the 24 Bytes EEG data and 2 Bytes CRC-16 checksum bits.

Figure S2. fNIRS signal processing workflow

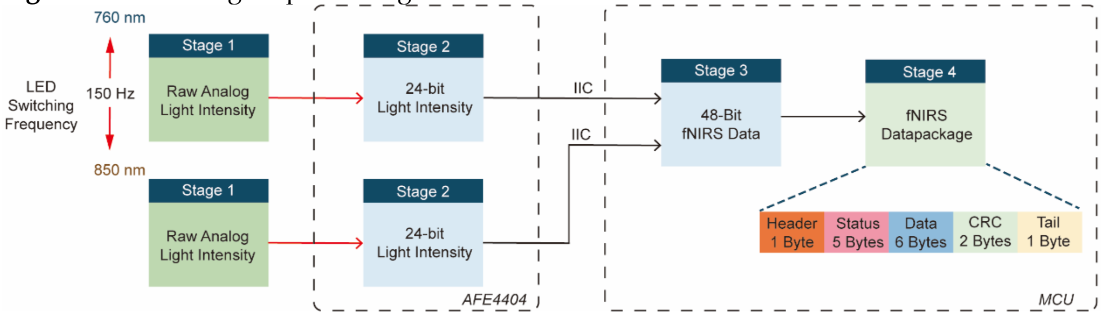

Raw current signal from PD is firstly converted to a voltage signal by the AFE4404's integrated TIA, and then digitized to 24 Bit light intensity data by the AFE4404's built-in 24 Bit ADC. AFE4404 also controls the emission wavelength of the dual-wavelength LED to switch between 760 nm and 850 nm at a certain frequency (150 Hz in the figure), and the light intensity data of the two neighboring different wavelengths will be transferred to the MCU through the IIC bus and combined into 48-bit light intensity data in the MCU. The MCU will packetize the fNIRS data, where the 1 Byte 'Header' and the 1 Byte 'Tail' are used to delimit the packets, the 5 Bytes 'Status' are used to indicate the packet serial number, packet length and data type, in addition to the 6 Bytes fNIRS data and 2 Bytes CRC-16 checksum bits.

Note S1. EEG and fNIRS data processing process in PC

The data packets are transmitted via ESP8285 Wi-Fi module to PC. The PC receives and decodes data packets using a self-designed controller based on C++ and Qt 5.14. The EEG data will be unpacked according to the EEG channel, and the fNIRS light intensity data will be unpacked according to the NIR wavelength and subtracted from the baseline light intensity data to obtain the optical density data, which will then be further computed as the concentration changes in oxy-, deoxy- and total hemoglobin using Matlab 2023a. (see section

3.3 in the main text for details)

Note S2. EEG acquisition performance evaluation method

Input-referred noise: Configure the CHnSET register in ADS1299 to enable the input shorted function. EEG Data is acquired at a sampling rate of 1kHz for 20 seconds. Input-referred noise is calculated as the root mean square voltage (V_{rms}) of the output signal.

Amplitude distortion: An SKX-8000C (Mingsheng) physiological signal generator was used to generate sine wave signals with an amplitude of 5, 10, 20, 30 and 50 μV with frequencies of 6 and 10 Hz respectively to the EEG signal inputs. EEG Data is acquired at a sampling rate of 1kHz for 20 seconds. Amplitude distortion (A) is calculated by Equation (1).

$$A = \frac{|V_o - V_i|}{V_i} \times 100\% \quad (1)$$

where V_o is the peak-to-peak voltage V_{pp} of the output signal, V_i is the amplitude of the input signal.

Frequency distortion: An SKX-8000C (Mingsheng) physiological signal generator was used to generate 5 groups of sine wave signals with an amplitude of 1000 μV at frequencies of 9, 11, 16, 20 and 30 Hz respectively to the EEG signal inputs. EEG Data is acquired at a sampling rate of 1kHz for 20 seconds. Frequency error (δ) is calculated by Equation (2).

$$\delta = \frac{|F_o - F_i|}{F_i} \times 100\% \quad (2)$$

where F_o is the frequency of the maximum amplitude in the spectrogram of output signal, F_i is the frequency of the input signal.

Note S3. Detailed experimental design for the event-related Stroop task

The stimulus program was coded using the Psychtoolbox-3 under MATLAB 2023a.

During the waiting period, the PC monitor displayed a pure black page.

During the task period, subjects were asked to perform the Stroop task. The Stroop task was used in which the Chinese words "红"(red), "绿"(green), or "蓝"(blue) were written in one of these three colors on a black background. The font size was 72 and was located in the center of the screen. If the color is green, subjects are asked to press the 'J' button on the keyboard. If the color is red, subjects are asked to press the 'K' button on the keyboard. If the color is blue, subjects are asked to press the 'L' button on the keyboard. The maximum reaction time of the subject is 500 ms. If the subject presses the correct key within the specified time, the screen will indicate "correct" and switch into the interval. If the subject pressed the wrong key or did not press the key within the reaction time, the screen will indicate "Error" and switch into the interval. During the interval, a white cross will be displayed in the center of the screen on a black background.

During the rest period, the PC monitor displayed a pure black page.
